# Sensory gating, inhibition control and gamma oscillations in the human somatosensory cortex

**DOI:** 10.1038/srep20437

**Published:** 2016-02-04

**Authors:** Chia-Hsiung Cheng, Pei-Ying S. Chan, David M. Niddam, Shang-Yueh Tsai, Shih-Chieh Hsu, Chia-Yih Liu

**Affiliations:** 1 Department of Occupational Therapy and Graduate Institute of Behavioral Sciences, Chang Gung University, Taoyuan, Taiwan; 2 Healthy Aging Research Center, Chang Gung University, Taoyuan, Taiwan; 3 Department of Psychiatry, Chang Gung Memorial Hospital, Taoyuan, Taiwan; 4 Institute of Brain Science, National Yang-Ming University, Taipei, Taiwan; 5 Brain Research Center, National Yang-Ming University, Taipei, Taiwan; 6 Graduate Institute of Applied Physics, National Chengchi University, Taipei, Taiwan; 7 Mind, Brain and Learning Center, National Chengchi University, Taipei, Taiwan; 8 Department of Medicine, Chang Gung University, Taoyuan, Taiwan; 9 Department of Traditional Chinese Medicine, Chang Gung University, Taoyuan, Taiwan

## Abstract

Inhibiting the responses to irrelevant stimuli is an essential component of human cognitive function. Pre-attentive auditory sensory gating (SG), an attenuated neural activation to the second identical stimulus, has been found to be related to the performance of higher-hierarchical brain function. However, it remains unclear whether other cortical regions, such as somatosensory cortex, also possess similar characteristics, or if such a relationship is modality-specific. This study used magnetoencephalography to record neuromagnetic responses to paired-pulse electrical stimulation to median nerve in 22 healthy participants. Somatosensory SG ratio and cortical brain oscillations were obtained and compared with the behavioral performance of inhibition control, as evaluated by somatosensory and auditory Go-Nogo tasks. The results showed that somatosensory P35m SG ratio correlated with behavioral performance of inhibition control. Such relationship was also established in relation to the auditory Go-Nogo task. Finally, a higher frequency value of evoked gamma oscillations was found to relate to a better somatosensory SG ability. In conclusion, our data provided an empirical link between automatic cortical inhibition and behavioral performance of attentive inhibition control. This study invites further research on the relationships among gamma oscillations, neurophysiological indices, and behavioral performance in clinical populations in terms of SG or cortical inhibition.

Inhibiting responses to irrelevant stimuli or inappropriate behaviors is an essential cognitive ability for humans in everyday life. For example, to precisely execute certain tasks in a distractible environment, task-relevant information is not only enhanced but task-irrelevant information is selectively inhibited at the same time. The Go-Nogo paradigms have been extensively used to study response inhibition processes^1^. In addition to attentive response inhibition, pre-attentive cortical inhibition is also of scientific and clinical importance, as it may be an indicator of central inhibitory function in patients who do not bear sustained attention or lack of cooperation during the examination.

Sensory gating (SG), or paired-pulse inhibition, referring to an attenuated neural response to the second identical stimulus, is considered as an automatic cortical inhibition function^2^,^3^. In the auditory modality, paired-click paradigms, in which two stimuli in close succession are presented, have been widely applied in the basic and clinical research^4^,^5^,^6^,^7^. It serves as a protective mechanism against flooding of the higher-order cortical centers with unnecessary information^4^,^8^. Although SG occurs at the early stage of perceptual processing, this mechanism may influence later cognitive processes. Previous studies have demonstrated a relationship between auditory SG and a variety of cognitive functions, such as visual attention^9^,^10^, visual-related memory task^11^, and visual-driven inhibition control^12^, suggesting a modality-independent relationship. However, up to the present, there is no empirical evidence to apply the same modality to elaborately assess the relationship between SG and behavioral response inhibition. Therefore, it remains an open question whether or not such relationships are modality-dependent or -independent. Besides auditory modality, SG has been also observed in the somatosensory cortical system^13^,^14^. Furthermore, it remains unknown whether pre-attentive SG in the somatosensory modality is also associated with behavioral performance of inhibition control.

Magnetoencephalography (MEG), providing excellent temporal resolution and reasonable spatial resolution, has been widely used to study sensory processes in the brain. Electrical stimulation to the median nerve activates the contralateral primary somatosensory cortex (SI), contralateral (SIIc) and ipsilateral (SIIi) secondary somatosensory cortices^15^,^16^,^17^,^18^. Paired-pulse electrical stimulation has previously been used to study the functional integrity of somatosensory cortical inhibition^14^,^19^,^20^,^21^. In addition to the time-domain waveforms, brain oscillations represent another aspect of brain function. Oscillations in the gamma frequency band (30 to 100 Hz) are modulated during various aspects of cognitive functions, including inhibition control^22^,^23^,^24^,^25^. Even in the resting state, it has been suggested that the peak value of gamma frequency, instead of power strength, in the visual cortex is closely related to the GABA concentration in the corresponding area^26^,^27^. Evidence also indicates that the generation of gamma oscillations is attributed to the balance setting of pyramidal cells and GABAergic inhibitory interneurons^28^,^29^. Through the link of GABA concentration between gamma oscillations and inhibition processes, it is reasonable to postulate that gamma oscillations in the somatosensory cortices may be associated with the SG ability and behavioral performance of response inhibition. However, there has been no study, to the best of our knowledge, to test this hypothesis.

More specifically, the goals of the present study were 3-fold. First, we sought to examine the somatosensory SG and its relation to the behavioral inhibition control. Second, we aimed to study whether the aforementioned potential link is modality-dependent or -independent. Third, due to the important role of gamma oscillations in the inhibition control, we further explored whether the peak value of gamma oscillations in the SI is associated with the somatosensory SG and performance of inhibition control. To these aims, somatosensory SG and gamma oscillations was recorded in the MEG laboratory, and auditory-driven and somatosensory-driven Go-Nogo tasks were assessed in the behavioral research laboratory.

## Results

**Figure 1:**
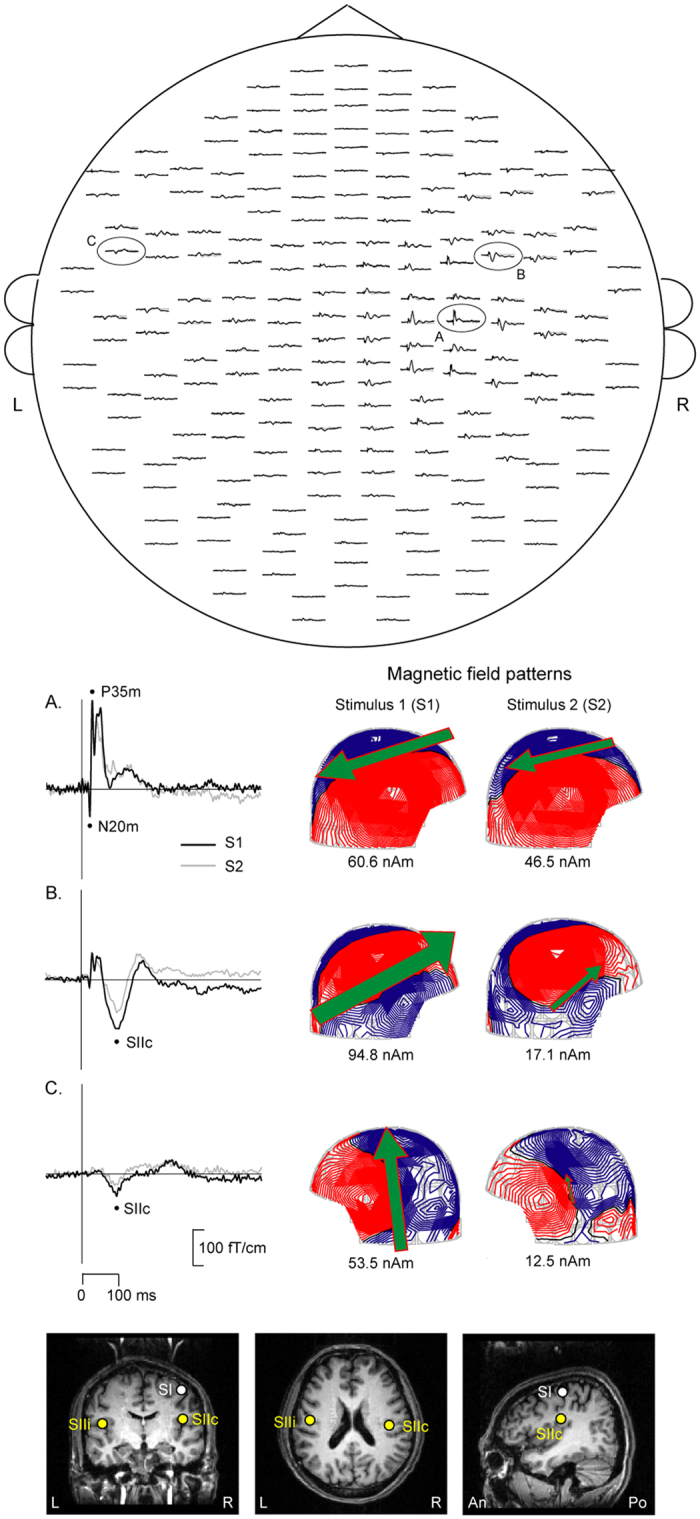
MEG signals for the paired-pulse electrical stimulation on the left median nerve in one representative subject. Upper panel: Spatial distribution of somatosensory evoked fields (SEFs) over 204 planar coils, viewed from the top of the head. Middle panel: The enlarged inserts from the encircled channels illustrate the magnetic field patterns at the peak latencies of the primary somatosensory cortex (P35m of SI, **A**), contralateral (SIIc, **B**) and ipasilateral (SIIi, **C**) secondary somatosensory cortex. Lower panel: The location of SEFs to Stimulus 1 on the MRI scans of the subject. R, right, L, left, An, anterior, Po, posterior.

Figure 1 demonstrates the spatial distribution of SEFs elicited by paired-pulse electrical stimulation of the left median nerve in a representative subject. In the human SI region, the N20m is the first deflection, followed by the P35m. The magnetic field patterns at the peak latencies of SI (P35m), SIIc, and SIIi are also presented. The lower panel shows the location of SEF sources superimposed on the magnetic resonance images (MRI) of the same subject. The P35m was located in the postcentral wall of the central fissure, and the generators of SII activity were located in the upper bank of the Sylvian fissure in the parietal operculum. In the time-domain analysis, the SG ratios of N20m, P35m, SIIc, and SIIi were 0.91 ± 0.05, 0.54 ± 0.03, 0.55 ± 0.03, and 0.35 ± 0.04, respectively. The time-frequency analysis revealed that the gating ratio of the gamma power in SI was 0.81 ± 0.07.

Table 1 shows the behavioral data in terms of accuracy rate of Go and Nogo stimuli, as well as the reaction time (RT) of correct Go trials. Subjects demonstrated a lower accuracy rate of Nogo stimuli compared to that of Go stimuli, for both the somatosensory-driven (Wilcoxon Sign Ranks Z = −3.68, *p* < 0.01) and auditory-driven (Wilcoxon Sign Ranks Z = −4.07, *p* < 0.01) tasks.

**Figure 2:**
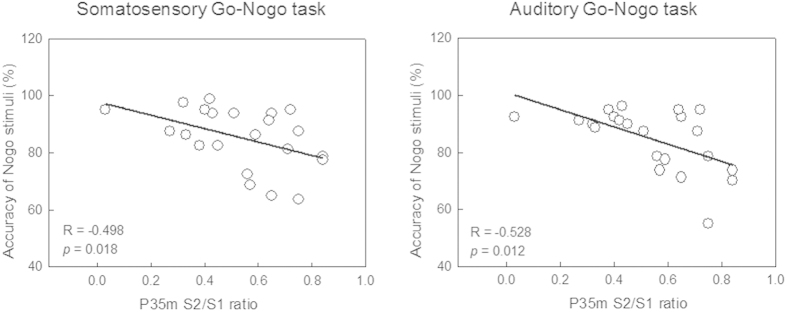
Correlation between cortical somatosensory P35m sensory gating (SG) ratio and accuracy rate of responses to Nogo stimuli. Somatosensory P35m SG ability is significantly associated with behavioral performance on inhibition control for both the somatosensory and auditory Go-Nogo tasks.

Table 2 shows the correlation coefficients of the relationship between somatosensory SG ratios and behavioral performance of Go-Nogo tasks. P35m S2/S1 ratios were significantly correlated with the accuracy rate of Nogo stimuli for both the somatosensory-driven (R = −0.498, *p* = 0.018), and auditory-driven (R = −0.528, *p* = 0.012) Go-Nogo tasks (Fig. 2). However, SG ratios of SIIc and SIIi were not significantly correlated with the performance of inhibition control.

**Figure 3:**
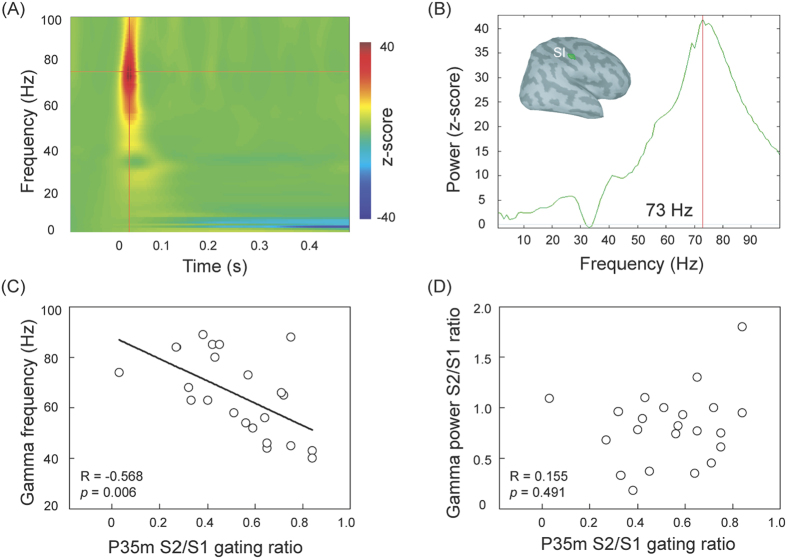
(**A**) Time-frequency maps of the primary somatosensory cortex (SI) region in the representative subject. A robust oscillatory activity peaks round 20 to 30 ms after the stimulus onset. (**B**) The power spectrum is derived from (**A**). The peak frequency value of the maximal gamma power is around 73 Hz. (**C**) The scatter plot, with best-fit linear regression, shows that the individual peak gamma frequency is correlated with P35m gating ratio. (**D**) There is no significant association between P35m and gamma power S2/S1 gating ratios in the SI region.

In the SI, robust evoked gamma oscillations were observed in all participants. Figure 3(A,B) exhibit the time-frequency map and the spectral responses of SI region for the same representative subject in Fig. 1. The peak frequency value of gamma oscillations showed considerable variability across all the participants, ranging from 40 to 89 Hz. Figure 3(C) reveals that the peak frequency value of the gamma oscillations was significantly and inversely correlated with somatosensory SG, as indexed by P35m S2/S1 ratio (R = −0.568, *p* = 0.006). However, P35m SG ratio was not associated with the power ratio (S2/S1) of evoked gamma oscillations in the SI, as shown in Fig. 3(D). We also did not observe significant relationship between peak frequency value of the gamma oscillations and behavioral performance of somatosensory-driven inhibition response.

## Discussion

This study examined the relationship between pre-attentive somatosensory SG ability by using MEG recordings and response inhibition through the evaluation of behavioral Go-Nogo tasks. Our results demonstrated that the somatosensory SG ability of the P35m component was correlated with the behavioral competence of inhibition control. Also, the aforementioned relationships were observed in the somatosensory-driven and auditory-driven Go-Nogo tasks, suggesting the association between SG and inhibition control was modality independent. In addition, by using time-frequency analysis from single trials, we found that a higher frequency value of evoked gamma oscillations in the SI was related to a better P35m SG ability.

SG has been considered as a protective mechanism to prevent higher-hierarchical centers from sensory overload^30^. It is, therefore, necessary to study whether pre-attentive SG ability correlates with the performance of cognitive function, such as attention, memory, and inhibition control. Wan and colleagues (2008) have reported a significant association of auditory P50 SG with performance on visual attention, as examined by the Attention Network Test and Stroop task^10^. Moreover, another study has demonstrated that a stronger auditory P50 gating ability correlated with fewer commission errors on the visual-driven Delayed Memory Task^11^, although some others have shown contradicting results^31^,^32^. With regards to the inhibition control, the Go-Nogo paradigm requires more inhibitory processes, and thus is more suitable to investigate the relationship between SG and inhibition execution. By applying this method, it has been shown that children with a stronger auditory P50 or N100 SG had a shorter RT in the visual Go-Nogo task^12^. Our current study, extending previous auditory event-related potential research, indicates that cortical somatosensory SG in SI is closely correlated with inhibition control as evaluated by the somatosensory Go-Nogo task, suggesting a better somatosensory SG relates to less commission errors. Such a relationship was, however, not found in the N20m of SI, SIIc and SIIi, despite an obvious gating ability in the SII areas. In the Go-Nogo experiment, the participants were required to respond to the electrical stimuli on the second digit as quickly and accurately as possible, and this rapid behavioral reaction or inhibition (if electrical stimuli were delivered to the fifth digit), might be primarily modulated by earlier cognitive operation occurring in the SI, rather than SII. A relatively larger variability of SII responses might also leads to a lower signal-to-noise ratio compared with the SI signals. With regards to the N20m, previous studies by using paired-stimulus paradigms have shown an almost complete recovery of N20m amplitude when an ISI of <100 ms was applied^33^,^34^. With the ISI of 500 ms in the current study, it was not surprised to find that N20m S2/S1 gating ratio nearly reached 1. Such the homogeneous values of SG ratio among the subjects fail to establish a significant relationship with behavioral performance.

With an established relationship between somatosensory P35m SG ability and behavioral performance on inhibition control, we further examined whether such an association corresponds to a modality-dependent or modality-independent manner. Previous studies have so far shown that the auditory SG ability was correlated with performance on visual-driven memory, attention, and response inhibition paradigms^10^,^11^,^12^. Our results extended the previous findings and, most importantly, provided empirical evidence to show that the somatosensory P35m SG ability was significantly correlated with both the somatosensory-driven and auditory-driven Go-Nogo tasks, suggesting a modality-independent relationship.

Brain rhythmic activity in the gamma range has been related to inhibition function. The link between higher gamma-frequency value and better inhibition response is potentially due to the intermedium of GABA^35^. It has been shown that the GABA levels decrease with the increment of age^36^. Furthermore, a positive association between peak value of gamma oscillations and GABA concentration was observed in the motor cortex^35^. Most importantly, there has been evidence showing that the intravenous injection of lorazepam, a GABA_A_-receptor agonist, modulated the somatosensory P35m SG ratio^19^. Therefore, the aforementioned data suggest a possible link between gamma-frequency value and SG ability, and our current results confirmed this notion. In this study, however, the value of the peak gamma oscillations in SI was only related to the somatosensory SG ability, but not to behavioral performance of somatosensory-driven inhibition execution. The current design of Go-Nogo paradigms might not be sensitive enough to directly reflect the neural activity. For example, Edden and colleagues (2009) showed a correlation of the peak frequency value of gamma oscillations only with oblique orientation threshold, but not with vertical orientation threshold^26^.

It is noteworthy that correlational neuroimaging data do not necessarily reflect a direct causal relationship. Although our research suggests that pre-attentive somatosensory SG might predict attentive inhibition control, further investigations using pharmacological interventions or virtual lesions induced by transcranial magnetic stimulation, for example, will provide important additional information on the causal mechanisms. With regards to methodological considerations, one might argue that the inter-pair interval of 6 s in the current experiment was not long enough for neuronal activation to return to baseline. However, our previous research, wherein the traditional inter-pair interval of 8 s was applied, revealed similar somatosensory P35m gating ratios, using either equivalent current dipole (ECD) modeling (around 0.58)^13^ or the minimum norm estimate method (around 0.61)^37^.

Several limitations should be addressed. First, we only collected the behavioral results from Go-Nogo tasks and compared them with the pre-attentive somatosensory SG ability. Nakata and colleagues established the somatosensory Go-Nogo protocols recorded by EEG or MEG, and provided convincing results of neural correlates of inhibition control, such as prefrontal cortex^38^,^39^,^40^. In addition, inferior frontal gyrus, supplementary motor area, anterior cingulate cortex, and inferior parietal cortex have been proposed to involve in the inhibition processing by using functional MRI^41^,^42^. It merits future research to obtain neuromagnetic responses to somatosensory Go-Nogo and paired-pulse paradigms to have a more in-depth examination of the relationships between SG and inhibition control. Second, in addition to gamma oscillations, the endogenous resting concentration of GABA, though not presented in the current study, has been hypothesized as a key property to reflect the inhibition mechanisms^27^,^43^. There is convincing evidence showing the association between GABA concentration and inhibition function in the visual cortex^26^, anterior cingulate cortex^44^, and prefrontal cortex^45^. It is important to extend previous findings to have further investigation on human inhibition mechanisms, from scopes of neurotransmitters, brain oscillations, to behaviors in the somatosensory system.

In summary, the current study demonstrated a correlation between the somatosensory SG ability and the performance competence of inhibition control, suggesting an association of the automatic cortical inhibition function with the attentive inhibition response. Furthermore, such a relationship was presented in either modality-dependent or modality-independent manner. Notably, the peak frequency value of evoked gamma oscillations in the SI was correlated with the somatosensory SG ability. These results might support further research on the relationships among gamma oscillations, neurophysiological indices, and behavioral performance in the clinical populations.

## Methods

### Participants

To exclude the potential impact of gender differences on the SG profile^46^, 22 healthy male volunteers (mean age 25.9 years old; rang 20–34 years old) participated in the present study. All subjects were right-handed and none had a history of neurological or psychiatric disorders. All refrained from smoking at least 12 hours before MEG recordings to avoid smoking-related confounding effects^47^. The study was approved by the Institutional Review Board of the Taipei Veterans General Hospital, with written informed consent obtained from all subjects. All experiments were performed in accordance with approved guidelines and regulations.

### MEG

Neuromagnetic recordings were made by using a whole-head 306-channel MEG (Vectorview, Elekta, Neuromag, Helsinki, Finland). The coil locations in relation to anatomical landmarks (nasion, left preauricular point, and right preauricular point) were determined with a 3D digitizer. The sampling rate was set at 1000 Hz with an online bandpass of [0.1, 200] Hz.

The left median nerve was stimulated at the wrist with 0.2 constant-current square-wave pulses by an electrical stimulator (Konstantstrom Stimulator, Schwind, Erlangen, Germany). The stimulus intensity was set at 20% above the motor threshold to elicit a visible twitch of the abductor pollicis muscles. Electrical stimulation was delivered in pairs with an inter-stimulus interval (ISI) of 500 ms, and an inter-pair interval of 6 s. The ISI of 500 ms allowed us to simultaneously examine the whole somatosensory system, including SI and bilateral SII regions^13^,^20^,^21^. During the somatosensory evoked field (SEF) recordings, subjects were instructed to focus on watching a silent movie they had selected to ignore the somatosensory stimulation. At least 100 trials for each stimulus type were collected for further analyses.

The averaged SEF data were offline filtered with a bandpass of [0.1, 120] Hz and a 100 ms pre-stimulus baseline correction. The source analysis was based on signals recorded by 204 gradiometers. Cortical sources of the SEFs were modeled using equivalent current dipoles (ECD) and a least-squares fit using subsets of 16–18 channels around the maximal responses. The goodness of fit value of the ECD model was estimated, and those values above 80% for the SI and 70% for the SII response were accepted for subsequent analyses. After identifying the individual dipoles in SI, SIIc, and SIIi, the ECDs were evaluated by a time-varying multiple-dipole model in which all the 204 gradiometers were taken into account^18^,^48^. The SG ratio was calculated according to the responses to Stimulus 2 (S2) over the responses to Stimulus 1 (S1) (S2/S1).

Although the evoked gamma oscillations might be superimposed on the N20m and/or P35m components regarding the time frame, the time-frequency responses provide additional information and solve the questions that SEFs could not answer^49^. Compared to the lower frequency bands, the somatosensory-evoked gamma oscillations represent the short-latency, early-stage information processing of the human SI. To assess the evoked gamma oscillations in the SI region, each raw single trial was analyzed by using Morlet wavelet-based time-frequency analysis from 1 to 100 Hz in 1 Hz steps (Brainstorm software^50^). The central frequency and time resolution were set at 1 Hz and 3 s, respectively. The peak value of gamma oscillations evoked by S1 and SG ratio of the gamma power strength (S2/S1) were calculated.

### Behavioral Go-Nogo tasks

The behavioral experiments were conducted after MEG recordings. The sequence of somatosensory-driven and auditory-driven Go-Nogo tasks were randomized and counterbalanced across participants. Each modality consisted of two blocks, each with 160 Go trials and 40 Nogo trials.

In the somatosensory-driven Go-Nogo task, stimuli (0.2 ms square wave current) were delivered to the second or fifth digit of the left hand. The stimulus intensity was 2.5 times the sensory threshold^39^. The anode was placed at the distal interphalangeal joint and the cathode at the proximal interphalangeal joint of the corresponding digit. Subjects were instructed to press the button with their right hand to stimuli on the left second digit (Go, 80%) as quickly and accurately as possible, and to withhold the responses to stimuli on the left fifth digit (Nogo, 20%). The ISIs varied between 1.0 and 1.2 s.

In the auditory-driven Go-Nogo task, the stimuli were delivered binaurally with 50 ms-duration sine-wave tones. Subjects were instructed to press the button with their right hand to the frequent low-pitched (800 Hz) tones (Go, 80%) as quickly and accurately as possible, and to withhold the responses to the infrequent high-pitched (850 Hz) tones (Nogo, 20%). The ISI also varied between 1.0 and 1.2 s, and the sound intensity was around 70 dB above the individual’s hearing threshold.

For each modality of the Go-Nogo tasks, the accuracy rate of Nogo stimuli was defined as [(trial number of successful inhibition/total Nogo stimuli) × 100%].

### Statistical analysis

All the data were presented as means ± standard error of the mean (SEM). To avoid spurious type I errors, nonparametric Spearman’s correlation coefficients were used to evaluate the relationships among SG, behavioral performance of inhibition control, and peak values of evoked gamma frequency bands. Two-tailed tests were conducted to determine the significant level, set as *p* values < 0.05.

## Additional Information

**How to cite this article**: Cheng, C.-H. *et al*. Sensory gating, inhibition control and gamma oscillations in the human somatosensory cortex. *Sci. Rep.* **6**, 20437; doi: 10.1038/srep20437 (2016).

## Figures and Tables

**Table 1:** Mean values (SEM) of behavioral Go-Nogo tasks.

	Go accuracy (%)	Nogo accuracy (%)	Go RT (ms)
Somatosensory	95.4 (1.5)	85.2 (2.2)*	355.3 (144.7)
Auditory	95.9 (1.1)	84.7 (2.3)*	371.4 (209.8)

*p < 0.001 compared with Go accuracy, SEM = standard error of the mean, RT = reaction time.

**Table 2:** Correlation coefficients between behavioral performance of inhibition control and somatosensory SG ratio.

	Somatosensory SG ratio					
	N20m	P35m	SIIc	SIIi	SI Gamma power	
Somatosensory Nogo accuracy (%)	0.062	−0.498*	0.207	0.007	0.171	
Auditory Nogo accuracy (%)	0.041	−0.528*	0.227	−0.070	−0.178	

*p < 0.05.

SG, sensory gating; SI, primary somatosensory cortex; SIIc, contralateral secondary somatosensory cortex; SIIi, ipsilateral secondary somatosensory cortex.
